# The ER Stress Surveillance (ERSU) pathway regulates daughter cell ER protein aggregate inheritance

**DOI:** 10.7554/eLife.06970

**Published:** 2015-09-01

**Authors:** Francisco J Piña, Maho Niwa

**Affiliations:** 1 Division of Biological Sciences, Section of Molecular Biology, Univeristy of California, San Diego, San Diego, United States; Howard Hughes Medical Institute, University of California, Berkeley, United States

**Keywords:** protein aggregates, ER stress, ER inheritance, ER Stress Surveillance, unfolded protein response, asymmetric cell division, *S. cerevisiae*

## Abstract

Stress induced by cytoplasmic protein aggregates can have deleterious consequences for the cell, contributing to neurodegeneration and other diseases. Protein aggregates are also formed within the endoplasmic reticulum (ER), although the fate of ER protein aggregates, specifically during cell division, is not well understood. By simultaneous visualization of both the ER itself and ER protein aggregates, we found that ER protein aggregates that induce ER stress are retained in the mother cell by activation of the ER Stress Surveillance (ERSU) pathway, which prevents inheritance of stressed ER. In contrast, under conditions of normal ER inheritance, ER protein aggregates can enter the daughter cell. Thus, whereas cytoplasmic protein aggregates are retained in the mother cell to protect the functional capacity of daughter cells, the fate of ER protein aggregates is determined by whether or not they activate the ERSU pathway to impede transmission of the cortical ER during the cell cycle.

**DOI:** http://dx.doi.org/10.7554/eLife.06970.001

## eLife digest

Many species of yeast form new cells by a process known as budding in which a small daughter cell ‘buds’ out of a larger mother cell. Mothers can only produce a limited number of buds before they die of old age. However, age is reset in the daughters to ensure that they are fully rejuvenated when born. Therefore, the mother cell needs to prevent the factors that cause aging and cell damage from entering the daughter.

Inside cells, proteins are made and folded correctly in a structure called the endoplasmic reticulum. If proteins are not folded properly, they are normally rapidly destroyed. However, if a cell requires lots of proteins to be made quickly, this can sometimes overwhelm and ‘stress’ the endoplasmic reticulum. When this occurs, proteins start misfolding and clump up to form toxic aggregates, some of which collect inside the endoplasmic reticulum.

The Endoplasmic Reticulum Stress Surveillance (ERSU) pathway monitors the health of the endoplasmic reticulum and prevents ‘stressed’ endoplasmic reticulum from entering daughter cells, which can cause them to die. By visualizing the endoplasmic reticulum and the aggregates contained within it during budding in the yeast species *Saccharomyces cerevisiae*, Piña and Niwa have now found that the ERSU pathway can also prevent these aggregates from entering daughter cells. However, if the ERSU pathway is not switched on—as may be the case if the level of endoplasmic reticulum stress is very low—then aggregates can enter the daughter cells. This is in contrast to protein aggregates that form elsewhere in the cell, which are normally always kept inside the mother cell due to their damaging effects.

These results suggest that the ERSU pathway is responsible for preventing protein aggregates in the endoplasmic reticulum from entering daughter cells, but only does so when these aggregates stress the endoplasmic reticulum. Future research will aim to identify how the ERSU pathway senses protein aggregates and prevents the transmission of damaged endoplasmic reticulum.

**DOI:** http://dx.doi.org/10.7554/eLife.06970.002

## Introduction

Asymmetric cell division is a mechanism that generates cells with different properties. Specifically, in *Saccharomyces cerevisiae*, asymmetric cell division allows daughter cell rejuvenation while ensuring that cellular damage is left behind in the mother cell (Henderson and Gottschling, 2008; Kaganovich et al., 2008; Spokoini et al., 2012; Gallagher et al., 2014; Nystrom and Liu, 2014; Zhou et al., 2014). Recent studies have revealed that cytoplasmic protein aggregates are retained in the mother cell, although the underlying mechanism(s) that establishes such an asymmetric mode of inheritance remains to be fully elucidated (Abbas et al., 2013). Furthermore, little is known about whether such asymmetric division is regulated during the cell cycle.

The endoplasmic reticulum (ER) is a gateway for the secretory pathway in eukaryotic cells. Proteins that are secreted or reside within the organelles of the secretory pathway initiate their journey when they are translocated into the membrane or lumen of the ER. In the unique oxidizing environment of the ER, nascent polypeptides undergo chaperone assisted folding and modifications, such as glycosylation and formation of disulfide bonds, to become mature active proteins before exiting from the ER (Mori, 2000; Rutkowski and Kaufman, 2004; Ron and Walter, 2007). In addition, the ER is a major site for lipid synthesis and storage of intracellular calcium (Mcmaster, 2001). Many of these ER functions must work in concert to satisfy cellular demands (Oakes and Papa, 2014). The unfolded protein response (UPR)-signaling pathway is a conserved response to ER stress and plays a critical role in maintaining ER function by up-regulating the transcription of genes coding for ER chaperones and protein-folding components (Cox et al., 1993; Mori et al., 1993; Ron and Walter, 2007; Wu et al., 2014). Importantly, the ER cannot be synthesized *de novo* and is generated only from existing ER. Given the critical function of the ER, it seems likely that cell cycle regulatory mechanisms must exist to ensure inheritance of a fully functional ER during cell division.

Recently, we reported the existence of a cell cycle surveillance mechanism or ‘checkpoint’ in *S. cerevisiae* that safeguards the inheritance of functional ER by the daughter cell (Bicknell et al., 2007; Babour et al., 2010). Upon ER stress induction, activation of this ER Stress Surveillance (ERSU) pathway results in re-localization of the cytokinesis-associated septin complex away from the bud neck, leading to a block in ER inheritance and cytokinesis. We showed that the ERSU pathway is independent of the UPR and is mediated by the Slt2 Mitogen-Activated Protein Kinase (MAPK). In the absence of Slt2, cells do not exhibit the block in ER inheritance and the septin ring remains at the bud neck following exposure to ER stress, similar to normally dividing, unstressed cells. Ultimately, however, *slt2Δ* cells are not able to sustain their growth due to the transmission of the stressed ER into the daughter cell. In fact, preventing ER transmission into *slt2Δ* daughter cells by genetic or pharmacological inhibition of actin polymerization can restore growth. Importantly, while Slt2 MAPK is known to play a role in the cell wall integrity (CWI) pathway, we found that the ERSU and CWI pathways are completely distinct (Babour et al., 2010; Levin, 2011). The discovery of the ERSU pathway thus not only identified a novel cell cycle checkpoint that ensures the inheritance of functional ER but also raised a number of important questions about the underlying mechanisms.

Furthermore, it is also unclear how the ER contents, including misfolded proteins, are segregated during the cell cycle. Under normal growth conditions, terminally misfolded proteins in the ER are retro-translocated into the cytoplasm and degraded by proteasomes in a process known as ER-associated degradation (ERAD) (Hampton, 2002; Bukau et al., 2006; Vembar and Brodsky, 2008; Smith et al., 2011; Thibault and Ng, 2012). When misfolded ER proteins are overexpressed or the ERAD function is diminished, the damaged proteins accumulate into large foci within the ER lumen. A recent study proposed that these large ‘aggregate’-like foci are selectively retained in the mother cell via a mechanism that depends on the lateral ER diffusion barrier established by the septin ring at the bud neck (Clay et al., 2014). Such lateral diffusion barriers between the mother and daughter yeast cells have been proposed to play pivotal roles in preventing undesirable materials, such as protein aggregates, from transferring to the daughter cells. While the exact mechanisms that establish the mother–daughter diffusion barrier remain to be elucidated, the barrier was reported to be formed as soon as the new bud emerges and depends on the bud site selection component GTPase, Bud1 (Clay et al., 2014). This study thus presented an attractive model suggesting that ER protein aggregate inheritance is regulated similarly to that of large protein aggregates in the cytoplasm, such as Q-bodies, JUNQ (juxta-nuclear quality control compartment) and IPOD (insoluble protein deposit), which are actively retained in the mother to protect the daughter cell from toxicity of the protein aggregates (Kaganovich et al., 2008). However, a potentially unique feature of ER protein aggregate inheritance is that it could be affected by inheritance of the ER itself. To further our understanding of how ER protein aggregates are divided between mother and daughter cells, we investigated the distribution of ER protein aggregates in relation to the inheritance of the ER.

## Results

### ER inheritance drives the transmission of ER protein aggregates into the daughter cell

**Figure 1.**
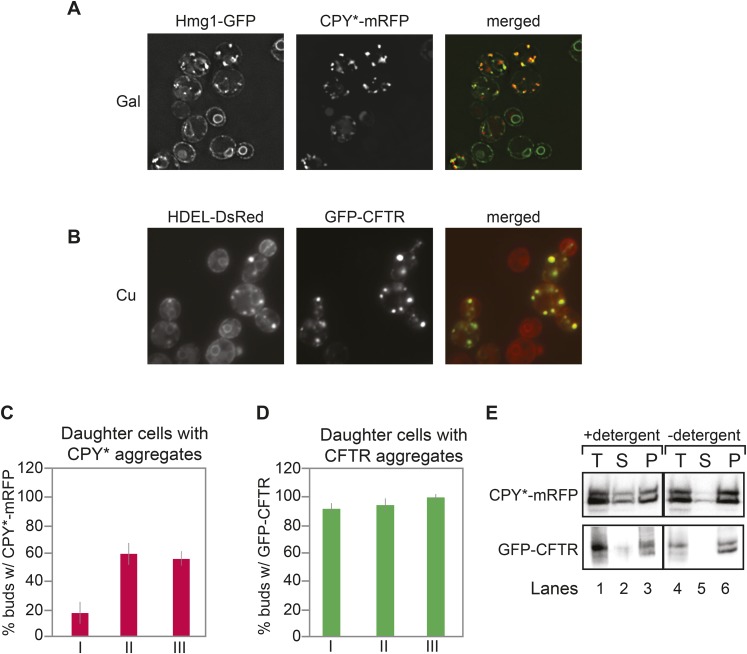
Inheritance of CPY* and CFTR aggregates by daughter cells. (**A**) Wild-type (WT) cells expressing galactose (Gal)-inducible CPY*-mRFP and the endoplasmic reticulum (ER) marker Hmg1-GFP were grown in 2% (w/v) Gal for 2 hr and then visualized by microscopy. Note that some Hmg1-GFP foci co-localized with CPY*-mRFP foci. (**B**) Cells expressing copper-inducible GFP-CFTR and the ER marker DsRed-HDEL were grown in copper-containing medium for 2 hr and then visualized. Note that DsRed-HDEL also co-localized with Cystic Fibrosis Transmembrane conductance Regulator (CFTR) foci. (**C** and **D**) Quantification of daughter cells containing CPY*-mRFP (**C**) or GFP-CFTR (**D**) foci at different stages of the cell cycle (small-budded cells, less than 2-µm length [class I]; medium-budded cells, greater than 2-µm length [class II], and large-budded cells containing nuclear ER [class III]). Error bars represent the standard deviation (SD) and were generated from at least three independent experiments with n > 100 cells. (**E**) CPY*-mRFP and GFP-CFTR foci are detergent insoluble and are present in the pellet fraction after detergent extraction. (T) Total, (S) supernatant, (P) pellet. These tests were previously utilized to characterize protein aggregates in cells (Alberti et al., 2010) and thus, we termed CPY*-mRFP and GFP-CFTR foci as aggregates throughout our study. **DOI:** http://dx.doi.org/10.7554/eLife.06970.003

To investigate the distribution of both the ER and ER protein aggregates between the mother and daughter cell, we monitored the distribution of a mutant form of the vacuolar protein carboxypeptidase Y (CPY*) fused to mRFP in cells also expressing Hmg1-GFP, a well-characterized ER marker (Finger et al., 1993; Nishikawa et al., 2001; Spear and Ng, 2005; Clay et al., 2014). A single amino acid change in CPY* (G255R) leads to improper folding, and the protein accumulates in the ER (Finger et al., 1993). Expression of CPY*-mRFP was placed under the control of the galactose (GAL1) promoter and induced by incubation in galactose-containing media. After 2 hr of induction, CPY*-mRFP formed aggregate-like foci that co-localized with both the cortical ER (cER) and perinuclear ER (pnER) (Figure 1A). We quantitated and evaluated the number of CPY*-mRFP foci in individual cells according to the daughter cell (bud) size (Figure 1C). A small number (<20%) of cells with small bud size (less than 2 μm in length; classified as class I cells (Babour et al., 2010), transferred CPY* foci to the bud, while the majority of cells (∼80%) contained foci only in the mother (Figure 1C). In contrast, ∼50–60% of class II (medium sized buds, larger than 2 μm in length) and class III (large buds with the nucleus and pnER in the bud) cells transferred CPY* foci to the bud (Figure 1C).

We also examined the inheritance of Cystic Fibrosis Transmembrane conductance Regulator (CFTR), which has been shown to form foci within the yeast ER (Fu and Sztul, 2003). In mammalian cells, a large proportion of newly synthesized wild-type (WT) CFTR is not properly folded and is ultimately degraded (Lukacs et al., 1994; Ward and Kopito, 1994; Jensen et al., 1995; Ward et al., 1995; Moyer et al., 1998; Gnann et al., 2004; Younger et al., 2006; Turnbull et al., 2007). Similarly, only a minor fraction of translated CFTR actually reaches the plasma membrane in yeast (Kiser et al., 2001; Zhang et al., 2001). GFP-CFTR foci were observed in the ER of cells expressing another well-characterized ER marker, DsRed-HDEL (Figure 1B). Significantly, most daughter cells, regardless of their class, inherited GFP-CFTR foci (Figure 1B,D).

Foci formed after expression of CPY* in yeast are often referred to and treated as protein aggregates without biochemical characterization. Therefore, we subjected CPY* foci to a well-established detergent extraction test commonly used to characterize protein aggregates (Alberti et al., 2010). The crude cell extracts prepared from CPY*-mRFP- or GFP-CFTR-expressing cells were treated with or without detergent and then fractionated by differential centrifugation. The majority of CPY*-mRFP was found in the insoluble protein pellet fraction regardless of detergent pre-treatment (Figure 1E). Similar results were obtained for GFP-CFTR (Figure 1E). These data indicate that the CPY* and CFTR foci observed here meet the definition of protein aggregates according to previous studies (Simons et al., 1995; Sondheimer and Lindquist, 2000; Nishikawa et al., 2001; Alberti et al., 2010), and we therefore refer to CPY*-mRFP and GFP-CFTR foci as aggregates throughout this study.

**Figure 2.**
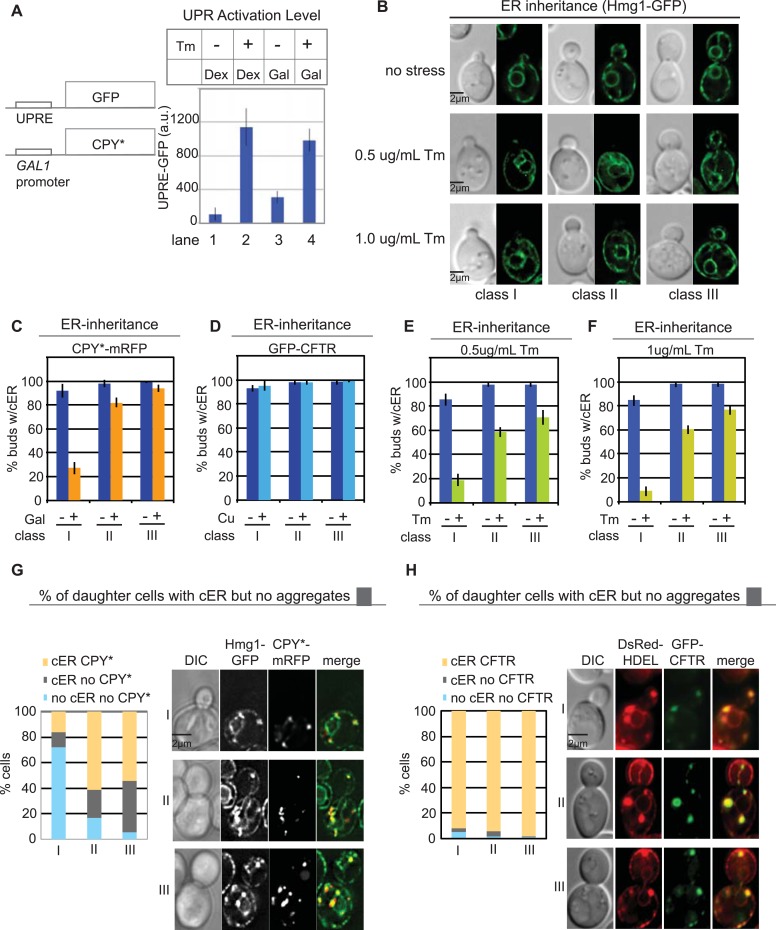
Differential inheritance of CPY* and CFTR aggregates and cER by daughter cells. (**A**) CPY*-mRFP expression activates the unfolded protein response (UPR) pathway. WT cells expressing CPY*-mRFP and the UPR reporter UPRE-GFP were incubated alone or with 1.0 μg/ml Tm, and GFP expression was quantified in individual cells. N > 100 cells per experiment; error bars (SD) were generated from at least three independent experiments. Dex: dextrose control medium, Gal; galactose-containing medium. (**B**) Quantitation of cortical ER (cER) in the buds of unstressed cells or cells treated with 0.5 or 1.0 μg/ml Tm for 3 hr cER inheritance was grouped by bud size: small-budded cells (class I), medium-budded cells (class II), and large-budded cells containing nuclear ER (class III). (**C**) CPY*-mRFP expression for 2 hr blocks cER inheritance. (**D**) GFP-CFTR expression for 2 hr in copper-containing media does not block cER inheritance. (**E** and **F**) Exposure to mild ER stress with 0.5 μg/ml Tm (**E**) blocks cER inheritance (induces the ER Stress Surveillance (ERSU) pathway) to a similar extent as does 1.0 μg/ml Tm (**F**). (**G**) Distribution (%) of cells at different stages of the cell cycle in which the daughter cells contain both cER and CPY*-mRFP aggregates (yellow), cER but not CPY*-mRFP aggregates (gray), and neither cER nor CPY*-mRFP aggregates (pale blue). Panel shows representative images of the most abundant cell types with CPY*-mRFP and Hmg1-GFP. (**H**) Same as **G** except that cells expressed GFP-CFTR and DsRed-HDEL. n > 100 cells were counted per experiment, repeated at least 3 times to generate error bars representing SD. **DOI:** http://dx.doi.org/10.7554/eLife.06970.004

Because both CPY*-mRFP and GFP-CFTR form aggregates in the ER, we tested whether the difference in their transmission to daughter cells might lie in the different effects of the aggregates on ER function. Previous studies have reported that expression of CPY*, but not CFTR, induces the UPR (Chaudhuri et al., 1995; Zhang et al., 2001). Indeed, we found that induction of CPY*-mRFP resulted in the expression of a UPR reporter (UPRE-GFP, Figure 2A, lane 3), and this was further increased upon treatment of cells with the glycosylation inhibitor tunicamycin (Tm), a well-characterized ER stress inducer (Figure 2A, lane 4) (Cox et al., 1993; Mori et al., 1993). We also asked whether CPY* aggregates activate the ERSU pathway, which functions to ensure the inheritance of functional cER (Babour et al., 2010). We found that a majority of the class I daughter cells did not inherit the cER under the ER stress condition evoked by CPY*-mRFP expression, and cER inheritance was also diminished in class II and III cells, but to a much lesser extent (Figure 2B,C). Additionally, the magnitude of the cER inheritance block caused by CPY*-mRFP expression was less than that induced by Tm treatment (Figure 2C,E,F). Finally, we found that 63% of class I, 73% of class II, and 60% of class III daughter cells containing the cER also contained CPY* aggregates (Figure 2G; yellow vs gray bars). Taken together, these data indicate that for CPY*-mRFP-expressing cells, more than 65% of buds that inherited ER also contained at least one CPY* aggregate (Figure 2—figure supplement 1A). Conversely, 57% of buds without aggregates also lacked the ER (Figure 2G, light blue vs gray).

**Figure 2—figure supplement 1.**
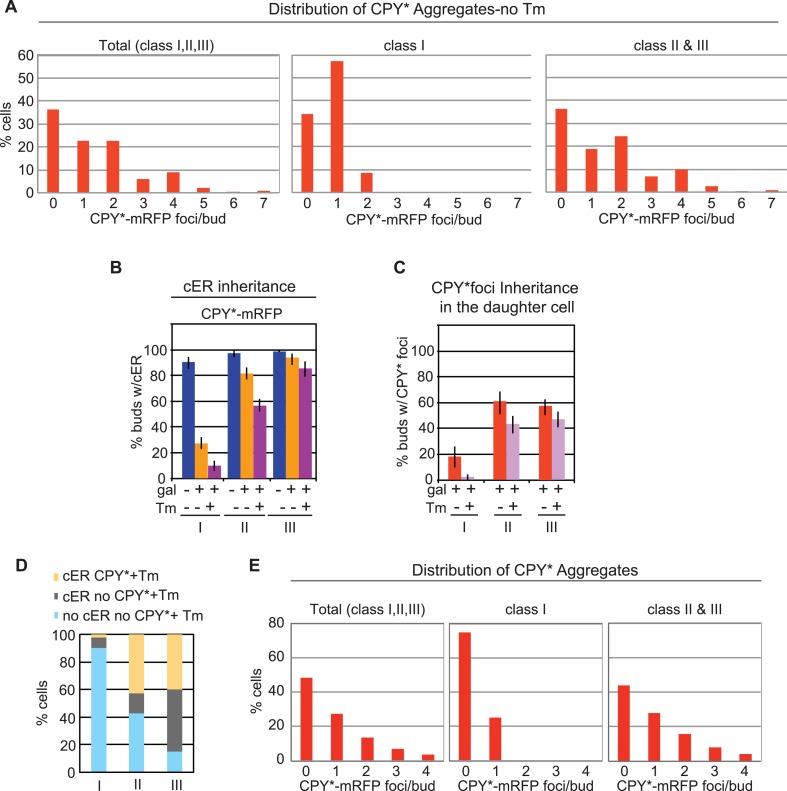
Inheritance of the ER and CPY* aggregates at different stages of the cell cycle. (**A**) Distribution of CPY*-mRFP aggregates per bud in all (Total), class I, and class II + III, and unstressed cells (without Tm). (**B**) cER inheritance by different classes of WT cells expressing CPY*-mRFP and left untreated or subjected to additional ER stress with Tm (1 μg/ml, 2 hr). (**C**) Percentage of daughter cells containing CPY*-mRFP aggregates following incubation with or without Tm. (**D**) Percentage of daughter cells containing cER and CPY*-mRFP aggregates (yellow), cER but no CPY*-mRFP aggregates (gray), and neither cER nor CPY*-mRFP aggregates (blue) for each class of cells. (**E**) Distribution of CPY*-mRFP aggregates per bud in all (Total), class I, and class II + III Tm-treated cells. **DOI:** http://dx.doi.org/10.7554/eLife.06970.005

**Figure 2—figure supplement 2.**
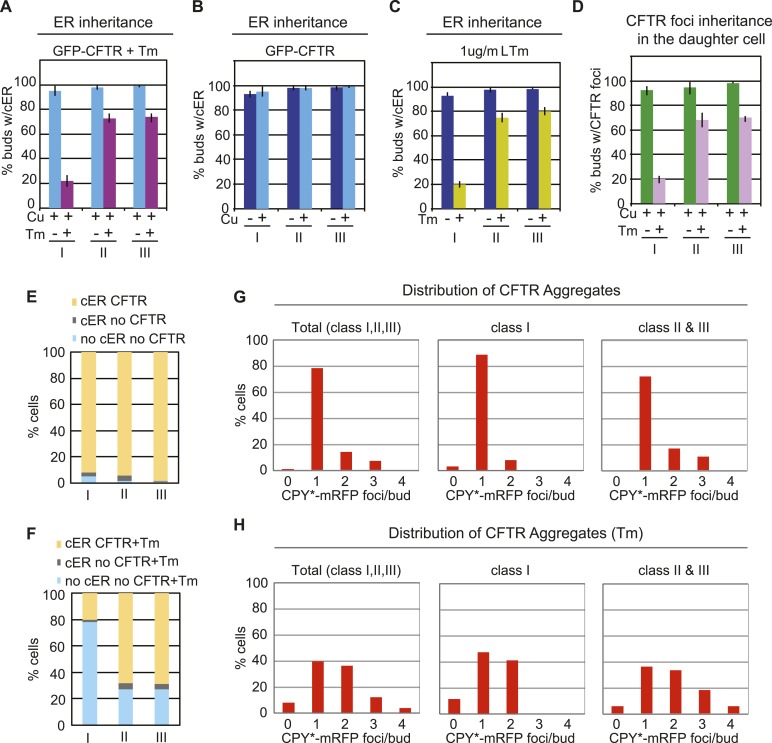
Inheritance of cER and CFTR aggregates at different stages of the cell cycle. (**A**) cER inheritance by different classes of WT GFP-CFTR-expressing cells treated without or with Tm (1 μg/ml, 2 hr). GFP-CFTR expression was induced by incubation in copper-containing medium (Cu). Graphs (**B**) and (**C**) are the same as those in Figure 2D,F but are shown again for comparison. (**D**) Percentage of daughter cells containing GFP-CFTR aggregates following incubation with or without Tm. (**E** and **F**) Percentage of daughter cells containing cER and GFP-CFTR aggregates (yellow), cER but no GFP-CFTR aggregates (gray), and neither cER nor GFP-CFTR aggregates (blue) for each class of cells. Cells were untreated (**E**) or treated with 1 μg/ml Tm (**F**). (**E**) The graph shown is the same as Figure 2H and is presented for comparison. (**G** and **H**) Distribution of GFP-CFTR aggregates per bud in all (Total), class I, and class II + III cells untreated (**G**) or treated with Tm (**H**). **DOI:** http://dx.doi.org/10.7554/eLife.06970.006

**Figure 2—figure supplement 3.**
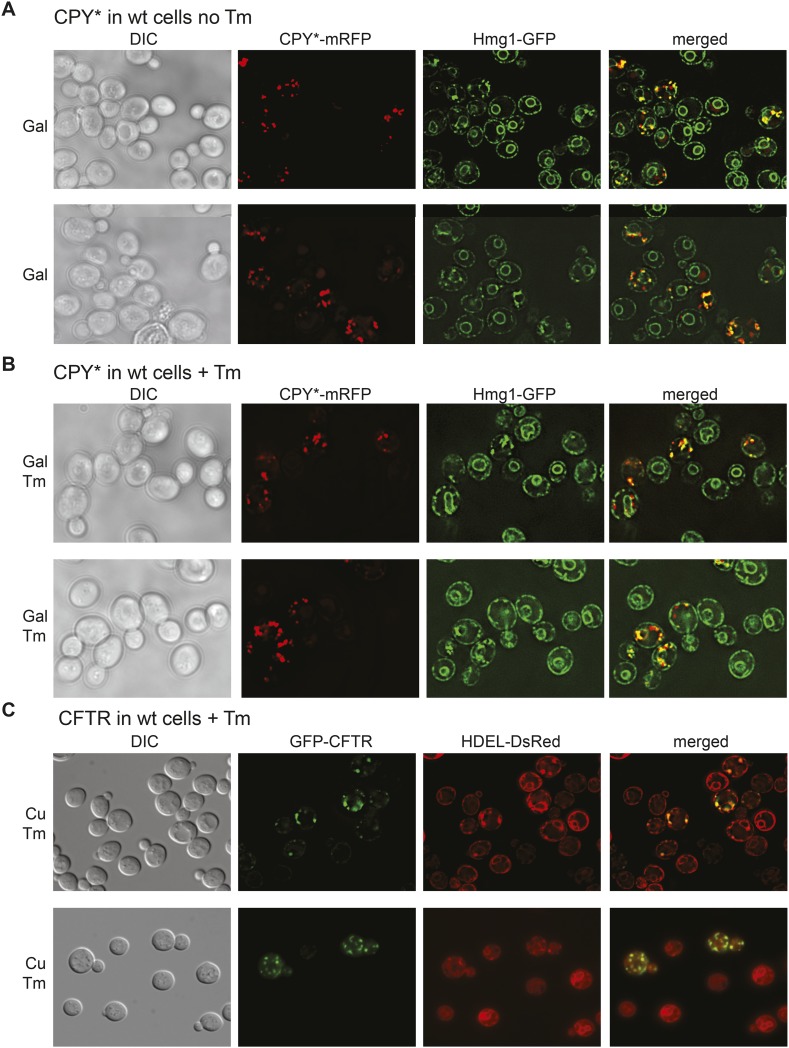
Colocalization of cER and CPY* or CFTR aggregates. (**A**) Two representative fields of cells expressing the ER marker Hmg1-GFP and treated with galactose (Gal) for 2 hr to induce expression of CPY*-mRFP. (**B**) Representative field of Hmg1-GFP-expressing cells treated with galactose (Gal) and Tm for 2 hr to induce expression of CPY*-mRFP. (**C**) Representative field of cells expressing the ER marker DsRed-HDEL and treated with 100 μM copper (Cu) and Tm for 2 hr to induce expression of GFP-CFTR. **DOI:** http://dx.doi.org/10.7554/eLife.06970.007

In contrast to the findings in CPY*-expressing cells, we found that cER inheritance was not affected by expression of GFP-CFTR (Figure 2D) unless the cells were also subjected to Tm treatment (Figure 2—figure supplement 2A–C). Therefore, CFTR aggregation presented an opportunity to evaluate the transmission of ER protein aggregates independently of the ER inheritance block. Quantitation of the number of CFTR aggregates and cER in the mother and daughter cells showed that virtually all class I, II, and III daughter cells contained CFTR aggregates (Figure 2H: yellow and Figure 2—figure supplement 2G), indicating no preferential retention of CFTR aggregates by the mother cells. Thus, although CPY* and CFTR both form ER protein aggregates in yeast cells, preferential retention of aggregates was only observed in the CPY*-expressing cells, which also displayed the cER inheritance block. Finally, the cER inheritance block and asymmetric distribution of CFTR aggregates was observed in GFP-CFTR-expressing cells after Tm treatment (Figure 2—figure supplement 2D–F,H), strengthening the relationship between ER inheritance and ER protein aggregate distribution.

### ER protein aggregate inheritance by daughter cells parallels the ER stress levels

Recently, it was reported that misfolded ER proteins were also retained in the mother cell when cells were exposed to relatively low levels of ER stress (0.5 μg/ml of Tm), as measured by Kar2sfGFP fluorescence recovery after photobleaching (FRAP) analysis (Clay et al., 2014). In our experiments, the moderate expression level of UPRE-GFP revealed that CPY*-mRFP aggregates induced a moderate level of ER stress (Figure 2A, lane 3) and also induced a mild block in ER inheritance compared with that induced by 0.5 μg/ml of Tm (compare Figure 2C,E,F, and Figure 2—figure supplement 3A). Based on the previous report, we anticipated that CPY*-mRFP expression alone (which induced low/medium levels of ER stress) should result in retention of CPY* aggregates in the mother cells. We observed, however, that CPY*-mRFP aggregates were distributed in both mother and daughter cells (Figure 2G, Figure 2—figure supplement 1A and Figure 2—figure supplement 3A). We also examined the effect of the compounded ER stress by treating CPY* aggregate-expressing cells with Tm (Figure 2—figure supplement 1B,E, and Figure 2—figure supplement 3B). As anticipated, the combined ER stresses further decreased ER inheritance by the daughter cells (Figure 2—figure supplement 1B). Notably, distribution of CPY* aggregates to the daughter cells was also further diminished in these cells (Figure 2—figure supplement 1C–E) and correlated with the reduced level of cER transmission (Figure 2—figure supplement 1B). Likewise, distribution of CFTR aggregates to the daughter cells was only diminished in cells in which ER inheritance was blocked by Tm treatment (Figure 2—figure supplement 2A,D, and Figure 2—figure supplement 3C). Collectively, these data therefore demonstrate that the magnitude of the ER inheritance block is affected by the level of ER stress, and that preferential retention of CPY* or CFTR aggregates in the mother cell is not an intrinsic property of the protein aggregates themselves, but rather, is dictated by ER inheritance.

### Aggregate inheritance is reduced in ERSU-deficient cells

**Figure 3.**
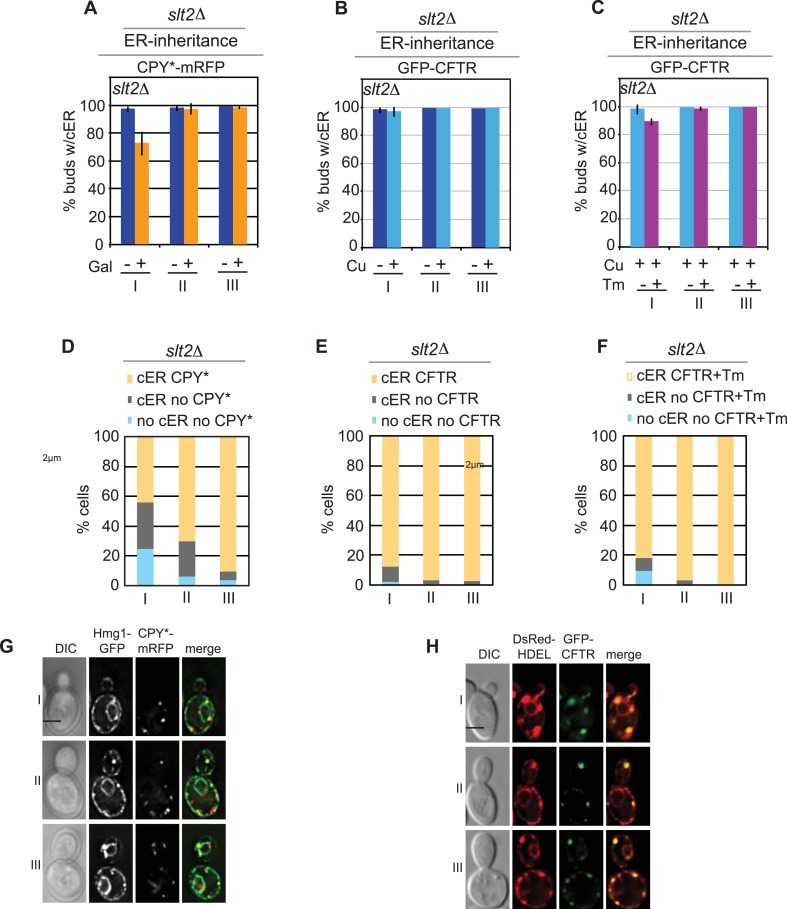
Inheritance of ER protein aggregates is ERSU dependent. (**A**) cER inheritance was not blocked in *slt2Δ* cells upon ER stress induction with CPY*-mRFP for 2 hr. (Compare to cER inheritance in CPY*-mRFP expressed WT cells [Figure 2C]). (**B**) GFP-CFTR expression for 2 hr has no impact on cER inheritance in *slt2Δ* cells. (**C**) GFP-CFTR expression for 2 hr in the presence of 1 µg/ml Tm does not block cER inheritance in *slt2Δ* cells. (**D**) Distributions (%) of *slt2Δ* cells that contain cER and CPY*-mRFP aggregates in daughter cells (yellow), cER but not CPY* aggregates (gray), and no cER and no aggregates (light blue) in different stages of cell cycle. (**E** and **F**) Distributions (%) of *slt2Δ *cells that contain cER and GFP-CFTR aggregates in daughter cells (yellow), cER but not GFP-CFTR aggregates (gray), and no cER and no aggregates (light blue) in different stages of cell cycle treated with (**F**) or without (**E**) Tm (1.0 µg/ml). (**G**) Representative images of *slt2Δ* cells in class I, II, and III with CPY*-mRFP and Hmg1-GFP to mark the ER. (**H**) Representative images of *slt2Δ* cells in class I, II, and III with GFP-CFTR and DsRed-HDEL ER marker. **DOI:** http://dx.doi.org/10.7554/eLife.06970.008

We reasoned that if the ERSU pathway-dependent ER inheritance regulates the distribution of protein aggregates to the daughter cell, then cells lacking the ERSU pathway should also show diminished retention of protein aggregates in the mother cell. To test this, we examined the distribution of CFTR and CPY* aggregates in *slt2Δ* cells, which are incapable of blocking ER inheritance in response to ER stress. We reported previously that Tm (1 μg/ml) treatment does not block cER inheritance in *slt2Δ* cells (Babour et al., 2010) and this was also observed in *slt2Δ* cells when ER stress was induced by CPY* aggregate expression (Figure 3A +Gal and Figure 3—figure supplement 1A; compare to Figure 2C for WT cells). Notably, entry of the cER into the daughter *slt2Δ* cells was paralleled by the entry of CPY* aggregates, which contrasts with the ERSU-dependent block in both cER and aggregate inheritance by WT daughter cells (Figure 3D,G, Figure 3—figure supplement 1A for *slt2Δ* vs 2G, Figure 2—figure supplement 3A for WT). As shown above, CFTR-expressing WT cells only exhibited a block in ER inheritance and CFTR aggregate transmission when treated with Tm (Figure 2—figure supplement 2A,D*,* and Figure 2—figure supplement 3C*:* Tm- vs Tm+). However, Tm treatment of CFTR-expressing *slt2Δ* cells failed to block either ER inheritance (Figure 3B,C, and Figure 3—figure supplement 1B vs Figure 3—figure supplement 1C) or CFTR aggregate entry into the daughter cell (Figure 3E,F,H, and Figure 3—figure supplement 1D). Taken together, these data revealed that ER inheritance, which is ultimately regulated by the ERSU pathway, regulates protein aggregate transmission into the daughter cell.

**Figure 3—figure supplement 1.**
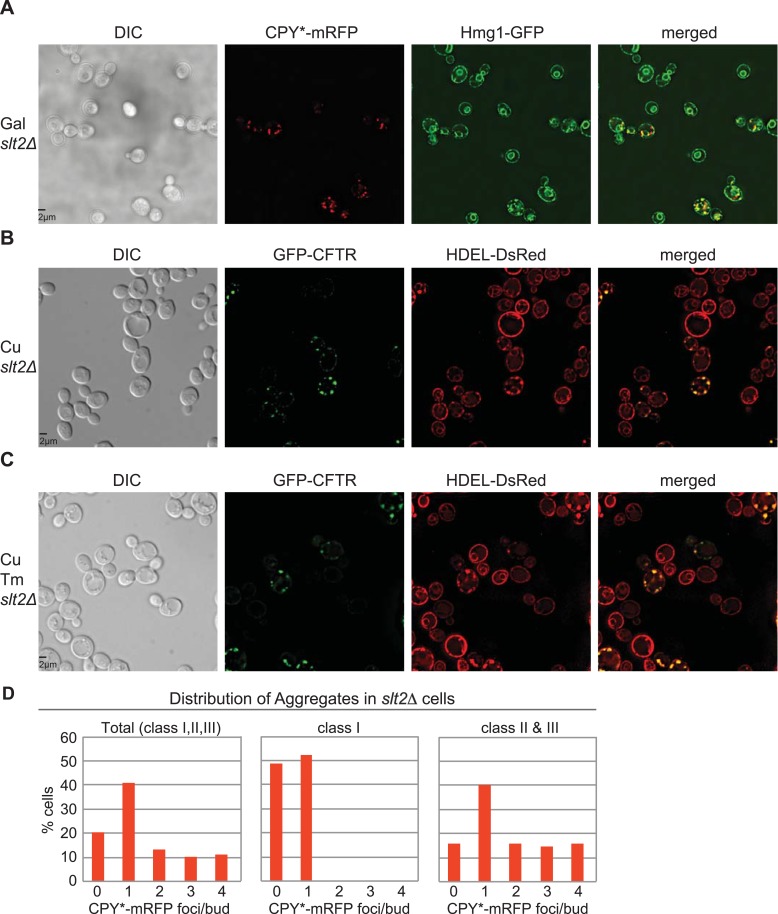
Colocalization of ER and CPY* or CFTR in *slt2Δ* cells. (**A**) Representative field of Hmg1-GFP-expressing *slt2Δ* cells treated with galactose (Gal) for 2 hr to induce expression of CPY*-mRFP. (**B**) Representative field of DsRed-HDEL-expressing cells treated with 100 μM copper (Cu) for 2 hr to induce expression of GFP-CFTR. (**C**) Representative field of DsRed-HDEL-expressing cells treated with 100 μM copper (Cu) and Tm for 2 hr to induce expression of GFP-CFTR. Note that *slt2Δ* cells do not exhibit a block in ER inheritance under ER stress. (**D**) Distribution of CPY*-mRFP aggregates per bud in all (Total), class I, and class II + III cells. **DOI:** http://dx.doi.org/10.7554/eLife.06970.009

### A Bud1-dependent diffusion barrier is not involved in the ERSU pathway or ER protein aggregate inheritance

**Figure 4.**
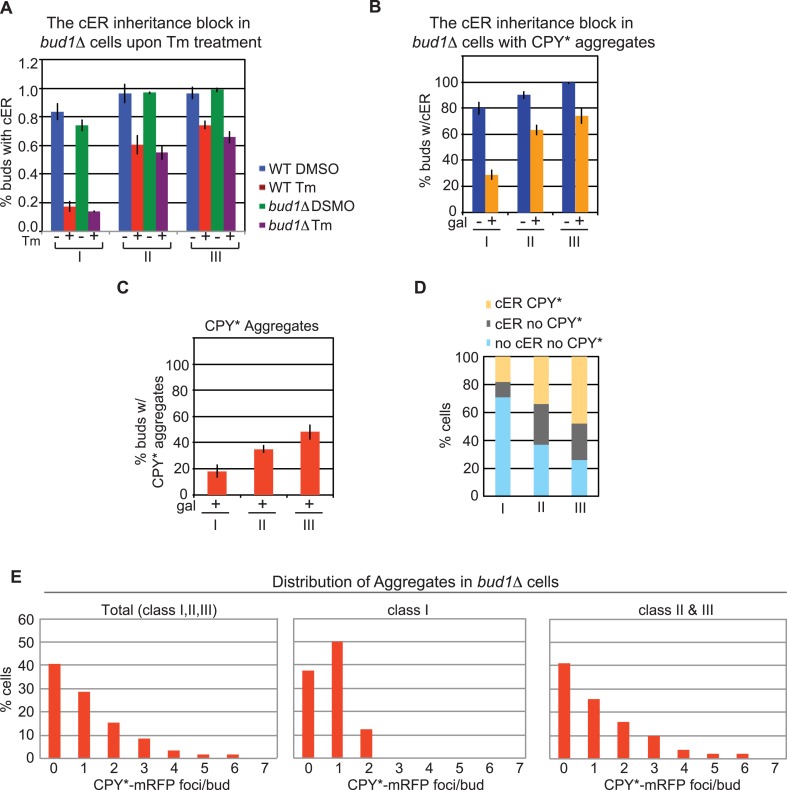
*BUD1* deletion has no effect on inheritance of the ER and CPY* aggregates. (**A**) cER inheritance is blocked to similar extents in WT and *bud1Δ* cells upon ER stress induction (1 μg/ml Tm for 3 hr). (**B**) *bud1Δ* cells display a normal block in ER inheritance upon exposure to ER stress induced by CPY*-mRFP expression for 2 hr. (**C**) Percentage of *bud1Δ* daughter cells containing CPY*-mRFP aggregates in class I, II, and III cells. (**D**) Distribution (%) of cells at different stages of the cell cycle in which daughter cells contain cER and CPY*-mRFP aggregates (yellow), cER but not CPY*-mRFP aggregates (gray), and neither cER nor CPY*-mRFP aggregates (light blue). (**E**) Distribution of CPY*-mRFP aggregates per bud in all, class I, and class II + III *bud1Δ* cells. **DOI:** http://dx.doi.org/10.7554/eLife.06970.010

As described in the introduction, previous work has suggested that a diffusion barrier limiting transmission of the cER between mother and daughter cells is formed during bud emergence via the activity of the Ras-like GTP-binding protein, Bud1 (Clay et al., 2014). Therefore, we examined whether Bud1 deficiency affected either cER inheritance or the distribution of ER protein aggregates. We found that the cER inheritance behavior of *bud1Δ* cells and WT cells subjected to ER stress by treatment with Tm (Figure 4A) or expression of CPY*-mRFP (Figure 4B) was similar. Furthermore, transmission of CPY* aggregates was also similar in the two strains (Figure 4C). These data suggest that Bud1 is not involved in the distribution of ER protein aggregates (see ‘Discussion’).

### Activation of the ERSU pathway is dependent on the cell cycle phase

**Figure 5.**
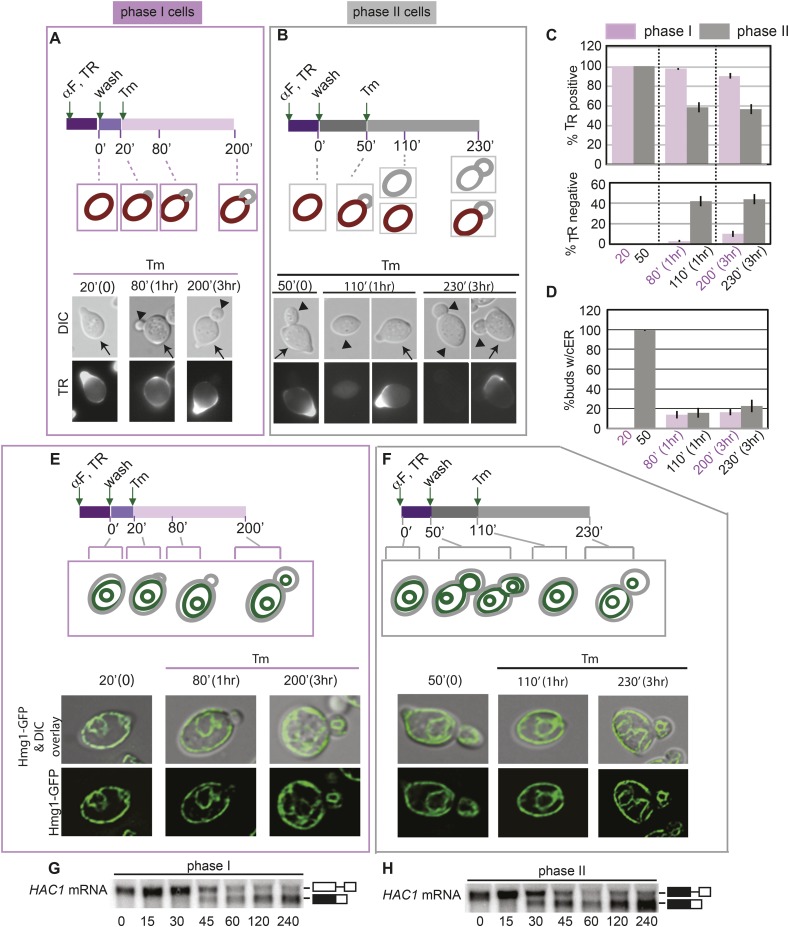
Activation of the ERSU pathway varies with the cell cycle stage. (**A**) ER stress was induced by treating synchronized WT cells (phase I: 20 min after α-factor release) with 1 μg/ml Tm. Cells were incubated with Texas Red (TR)-ConA during the α-factor treatment and then washed before exposure to Tm. Mother cells (TR-positive, arrows) can thus be distinguished from daughter cells emerging after induction of ER stress (TR-negative, arrowheads), as shown in the upper schematic. Cells were analyzed by DIC and fluorescence microscopy at the indicated times prior to and after addition of Tm. (**B**) As described for **A**, except that Tm was used to induce ER stress in phase II cells (50 min after α-factor release). Many cells underwent cytokinesis, as evident from the presence of unbudded TR-positive and TR-negative cells. (**C**) Quantification of TR-positive and TR-negative phase I (purple) and phase II (gray) cells at the time points indicated. (**D**) Quantification of cER inheritance by the daughters of phase I (**E**) and phase II (**F**) cells upon Tm treatment for the indicated times. (**E** and **F**) Hmg1-GFP-expressing phase I (**E**) and phase II (**F**) cells were treated as shown in **A** and **B**, and cER inheritance was evaluated at the indicated times. (**G** and **H**) UPR induction occurred regardless of the time of addition of Tm. *HAC1* mRNA splicing was measured as an indicator of UPR induction in phase I (**G**) and phase II (**H**) cells. Northern blotting of *HAC1* mRNA was performed at the indicated times after Tm treatment. Positions of the spliced and unspliced *HAC1* mRNA are indicated. **DOI:** http://dx.doi.org/10.7554/eLife.06970.011

In the experiments described above, we noted that a significant population of class II and III daughter cells contained the cER even after Tm treatment (Figure 2B–F). We considered that these cells might be incapable of inducing the ER inheritance block or that they may be daughter cells that had already inherited the cER before induction of ER stress. In both cases, one would expect that the retention of the stressed ER, and thus, of ER protein aggregates, in the mother cell would be limited only to cells with small daughter cells. To investigate these possibilities, we synchronized yeast cells in G1 by incubation with α-factor. After washing to remove α-factor, the cells were allowed to proceed normally through the cell cycle for either 20 min (Figure 5A; phase I cells) or 50 min (Figure 5B; phase II cells) before ER stress was induced by addition of Tm. To unambiguously identify the original mother cells present at the time of α-factor arrest, we fluorescently labeled cells by incubation with Texas Red (TR)-conjugated ConA (TR-ConA) during the α-factor treatment (Figure 5A,B). Thus, after washout of both α-factor and TR-ConA, newly emerging daughter cells will be TR-negative, while the mother cell remains TR-positive (Figure 5A–C, and Figure 5—figure supplements 2, 3).

**Figure 5—figure supplement 1.**
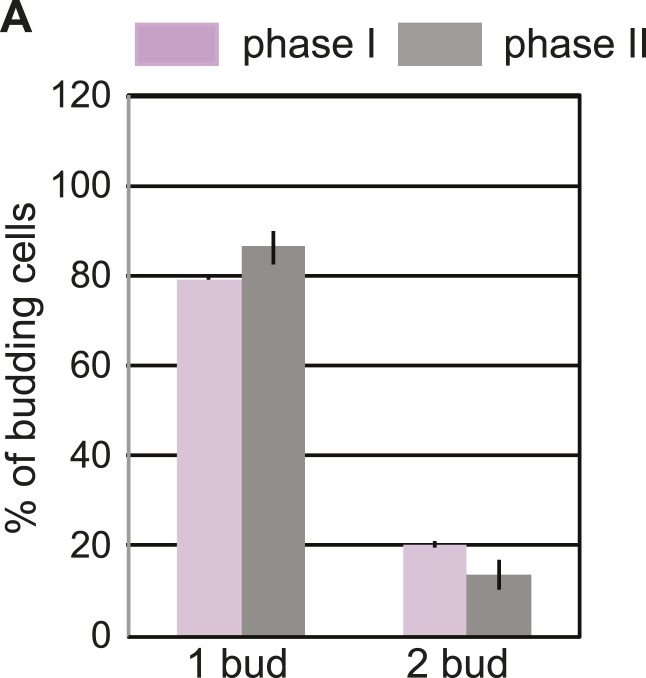
Phase I and phase II cells activate the ERSU pathway. (**A**) Percentage of phase I (purple) and phase II (gray) cells that showed one bud or two buds after Tm treatment. Error bars indicate SD from three independent experiments. **DOI:** http://dx.doi.org/10.7554/eLife.06970.012

**Figure 5—figure supplement 2.**
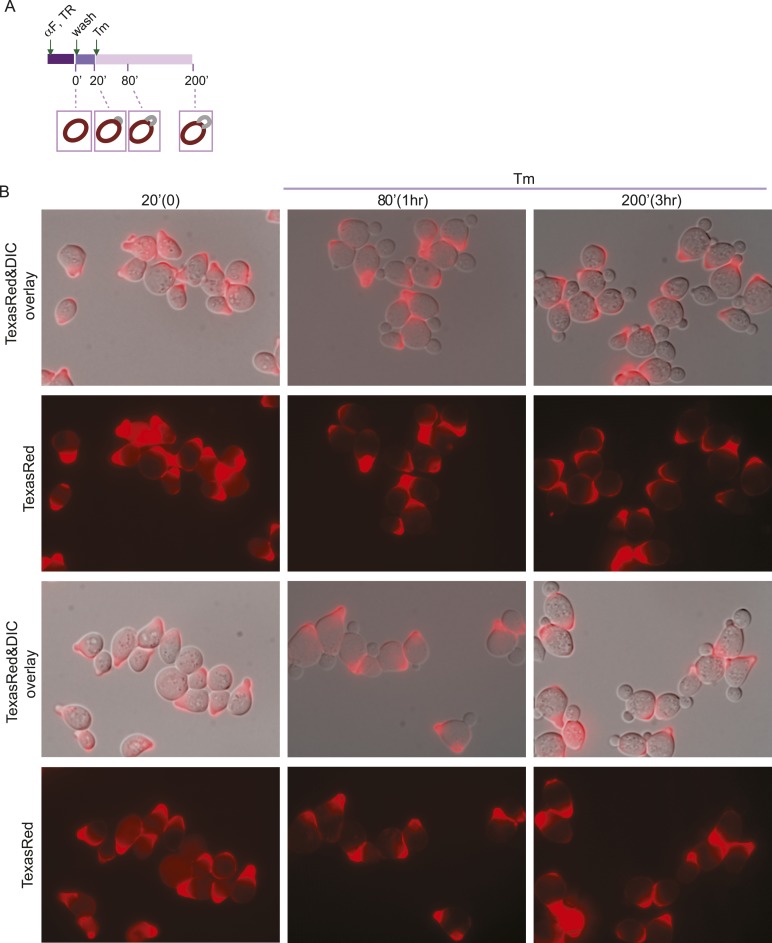
ER stress induction in cells at an early stage of the cell cycle. (**A**) A schematic diagram of the experimental set-up for examination of phase I cells. WT cells were synchronized with α-factor, washed, and treated with 1 μg/ml Tm 20 min later (phase I: 20 min after α-factor release). (**B**) Representative fields of cells treated as in (**A**) and incubated with Texas Red (TR)-ConA during the α-factor treatment. Mother cells (TR-positive) can thus be distinguished from daughter cells emerging after induction of ER stress (TR-negative). **DOI:** http://dx.doi.org/10.7554/eLife.06970.013

**Figure 5—figure supplement 3.**
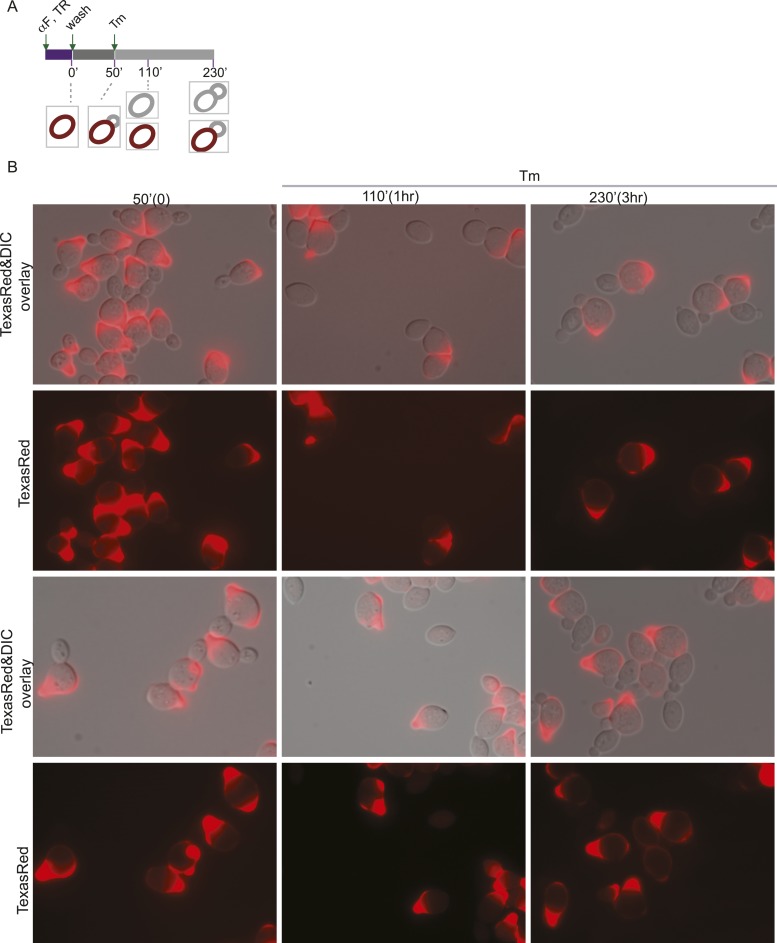
Activation of the ERSU pathway varies with the cell cycle stage. (**A**) A schematic diagram of the experimental set-up for examination of phase II cells. WT cells were synchronized with α-factor, washed, and treated with 1 μg/ml Tm 50 min later (phase II: 50 min after α-factor release). (**B**) Representative fields of cells treated as in (**A**) and incubated with Texas Red (TR)-ConA during the α-factor treatment. Mother cells (TR-positive) can thus be distinguished from daughter cells emerging after induction of ER stress (TR-negative). Many cells underwent cytokinesis, as evident from the presence of TR-positive and TR-negative cells at 110 and 230 min. **DOI:** http://dx.doi.org/10.7554/eLife.06970.014

**Figure 5—figure supplement 4.**
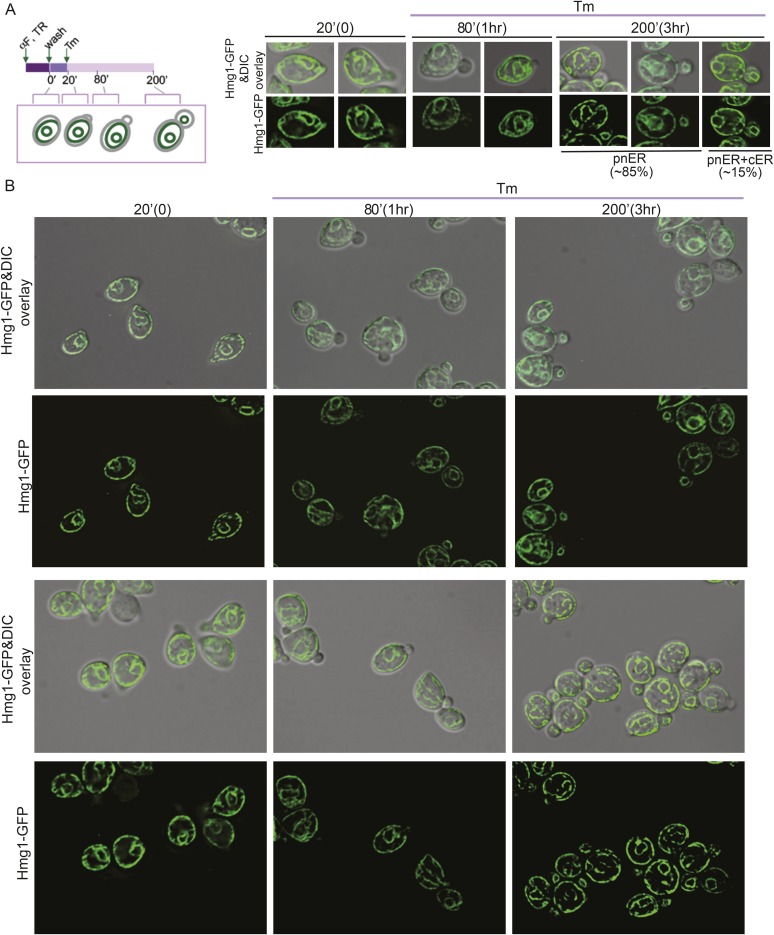
Activation of the ERSU pathway in cells at an early stage of the cell cycle. (**A**) A schematic diagram of the experiment shown in Figure 5E, shown again here for clarity. ER stress was induced by treating α-factor-synchronized Hmg1-GFP-expressing WT cells (phase I: 20 min after α-factor release) with 1 μg/ml Tm, and cER inheritance was evaluated at the indicated time points. Two representative cells at each time point are shown. For the 200 min time point (3 hr after addition of Tm), representative daughter cells containing only the pnER (85% of total cells) or both the pnER and cER (15% of total cells) are shown for comparison. Another set of representative cells is shown in Figure 5E. (**B**) Two representative fields of cells are shown for each time point. **DOI:** http://dx.doi.org/10.7554/eLife.06970.015

**Figure 5—figure supplement 5.**
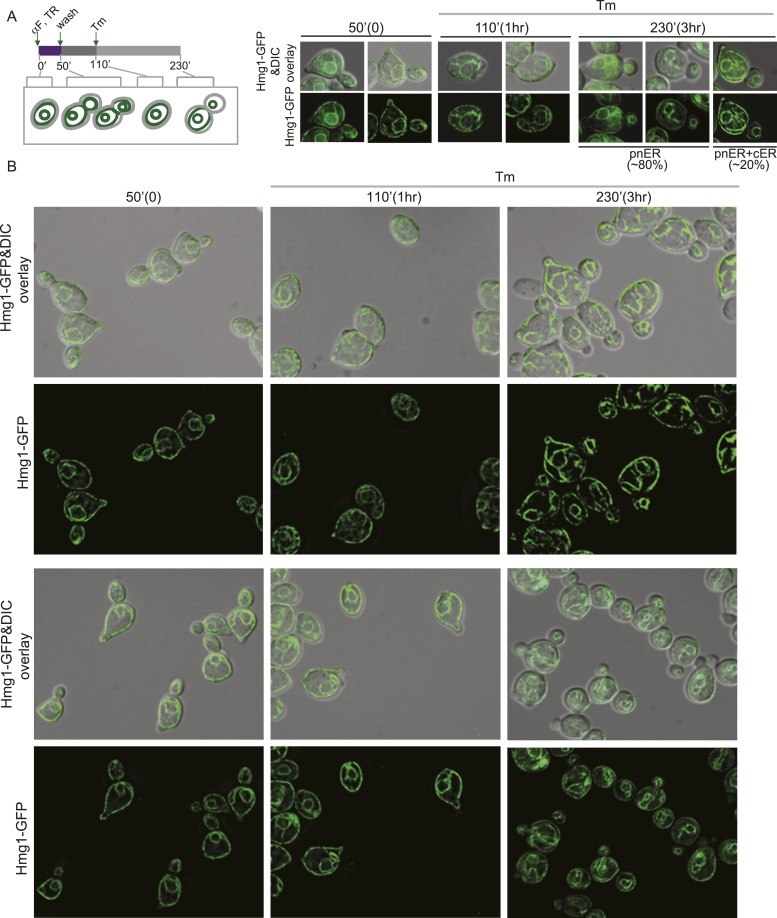
Activation of the ERSU pathway in cells at a later stage of the cell cycle. (**A**) A schematic diagram of the experiment shown in Figure 5F, shown again here for clarity. ER stress was induced by treating α-factor-synchronized Hmg1-GFP-expressing WT cells (phase II: 50 min after α-factor release) with 1 μg/ml Tm, and cER inheritance was evaluated at the indicated time points. Cells underwent cytokinesis, as evident from the presence of unbudded cells at 110 min (also see Figure 5B,C). Two representative cells at each time point are shown. For the 230 min time point (3 hr after addition of Tm), representative daughter cells containing only the pnER (80% of total cells) or both the pnER and cER (20% of total cells) are shown for comparison. Another set of representative cells is shown in Figure 5F. (**B**) Two representative fields of cells are shown for each time point. **DOI:** http://dx.doi.org/10.7554/eLife.06970.016

We found that phase I cells exhibited a cytokinesis block and did not undergo cell division at 80 min after α-factor release (1 hr after Tm addition; Figure 5A,C and Figure 5—figure supplement 2). Even at 200 min after release (3 hr post-Tm), >85% of phase I cells remained undivided (Figure 5A,C; purple bars and Figure 5—figure supplement 2). Furthermore, in most phase I cells, the cER remained in the mother cell (Figure 5D–E and Figure 5—figure supplement 4). In contrast, at 50 min after α-factor release (before induction of ER stress), almost all of the phase II daughter cells had already inherited the cER (Figure 5D,F and Figure 5—figure supplement 5). After Tm addition, these cells underwent cytokinesis (cell division), and at 1 hr after Tm addition (110 min), ∼50% of cells were derived from TR-positive mother cells, and the remaining ∼50% were TR-negative and were derived from the first daughter cell that emerged after α-factor release (Figure 5B,C, 110 min gray bars and Figure 5—figure supplement 3). After division, the number of cells with the cER in the daughter cell was small (Figure 5D,F; 110 min, and Figure 5—figure supplement 5). The observed differences between phase I and phase II cells in cytokinesis and ER inheritance were not due to an inability of phase II cells to respond to Tm. Phase I and II cells showed similar degrees of UPR activation after ER stress, as reflected in the levels of spliced *HAC1* mRNA resulting from activated Ire1 RNase-mediated mRNA cleavage (Figure 5G,H). Intriguingly, phase II cells exhibited a block in both cytokinesis and cER inheritance block at the second round of division, and the daughter cell arising from the first cell division did not further divide. Instead, we observed cells with two daughter cells. This was also observed for phase I cells in which the second daughter cell started to emerge after 3 hr of Tm treatment. At this point (Tm, 3 hr), ∼13% of phase II and ∼20% of phase I cells had two buds (Figure 5—figure supplement 1). These results therefore demonstrate that cells in which the cER is already in the daughter cell at the time of ER stress induction proceed through cytokinesis once, but display blocks in both cytokinesis and cER inheritance in the next cell cycle.

### Mother and daughter cells display similar levels of ER stress

**Figure 6.**
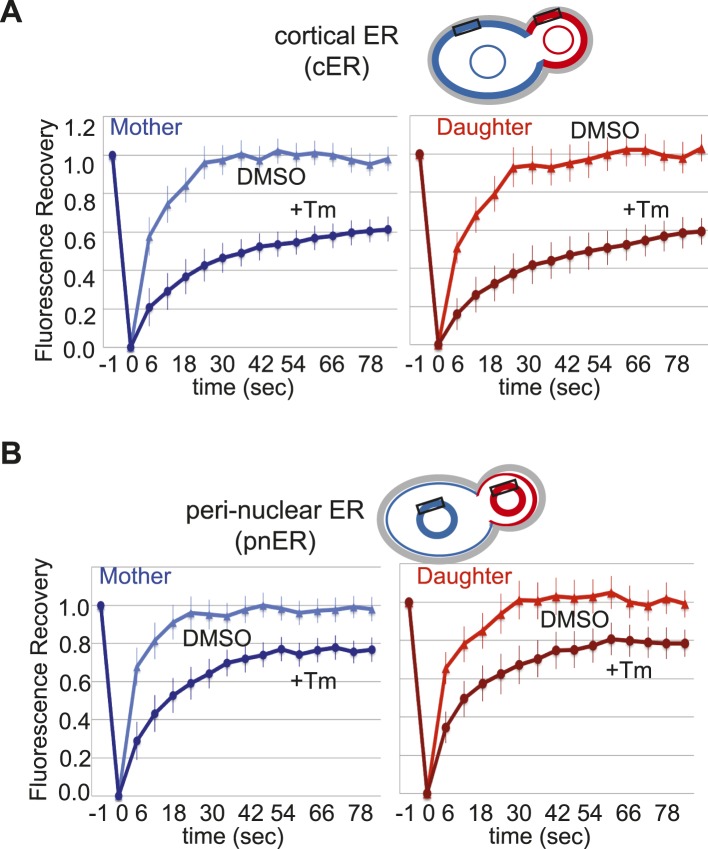
Mother and daughter cells display similar degrees of cortical and perinuclear ER stress. (**A**) Quantification of ER stress was performed by fluorescence recovery after photobleaching (FRAP) of cells labeled with the ER chaperone Kar2/BiP-sfGFP. Cells were exposed to DMSO or Tm (1 μg/ml) and then discrete regions of cER (indicated by the black rectangles) in mother (blue) and daughter (red) cells were photobleached and recovery was monitored. (**B**) Cells were treated as in **A**, except that FRAP was monitored in the indicated regions of the perinuclear ER (pnER; rectangles). cER and pnER stress, as indicated by the rate of FRAP, was comparable in untreated or Tm-treated mother and daughter cells. The results are the average of three independent experiments, each of which analyzed at least seven independent cells under both DMSO and Tm-treated conditions. **DOI:** http://dx.doi.org/10.7554/eLife.06970.017

Finally, we considered that if ER protein aggregates are preferentially retained in the mother cell independently of the ERSU pathway, then the ER stress levels in the mother cell should also be elevated relative to the daughter cells. To test this, we used a FRAP assay with WT cells expressing Kar2/BiP-sfGFP reporter (a major ER chaperone fused to ‘superfolder’ GFP), which displays significantly better folding in the oxidizing ER luminal environment than GFP or EGFP (Pedelacq et al., 2006; Lai et al., 2010; Aronson et al., 2011; Lajoie et al., 2012). In both mammalian and yeast cells, Kar2/BiP binding to unfolded client proteins increases in response to ER stress, reducing its mobility within the ER lumen (Snapp et al., 2006). This can be monitored by the reduced rate of FRAP. Small areas of the cER or pnER (indicated by black rectangles in Figure 6) in the mother and daughter cell were photobleached and the rate of Kar2/BiP-sfGFP fluorescence recovery from the surrounding area was assessed. In the mother cell, the fluorescence recovery rate was significantly reduced by Tm treatment when compared with control DMSO-treated cells, and this was similar for both the cER and pnER (Figure 6A,B). However, there were no significant differences between the mother and daughter cell in recovery rates in either the cER or pnER under control or Tm-treated conditions (Figure 6A,B), indicating that the ER stress levels are identical in mother and daughter cells. Taken together, the data presented do not support the preferential retention of ER protein aggregates in the mother cell, but instead argue that ER inheritance regulates the fate of unfolded proteins and the inheritance of ER protein aggregates by the daughter cell.

## Discussion

In budding yeast, the decision of whether or not to transfer specific cellular components or organelles to the daughter cell is critical for the health of the new generation. Limiting the transmission of potentially harmful components ensures that the functional capacity of the new daughter cell is reset (Shcheprova et al., 2008; Liu et al., 2010; Peraza-Reyes et al., 2010; Hughes and Gottschling, 2012; Longo et al., 2012; Ouellet and Barral, 2012). A recent report demonstrated that ER-resident misfolded protein aggregates, such as a mutant form of carboxypeptidase (CPY*), were prevented from entering the daughter cell and were retained within the mother cell (Clay et al., 2014). The expression of certain misfolded proteins or protein aggregates, including CPY*, in the ER lumen induces ER stress (Spear and Ng, 2003), as also shown here. In turn, ER stress activates the ERSU pathway, which blocks ER transmission into the daughter cell (Babour et al., 2010). These observations raise important questions about the potential mechanism(s) underlying the lack of CPY* aggregates in the daughter cell. One explanation is that transmission is blocked concomitantly with the ERSU pathway-mediated block in ER inheritance. Alternatively, independent regulatory mechanisms may also control the segregation of ER protein aggregates. The results of our experiments described here unambiguously demonstrated that the ERSU pathway governs the location of ER protein aggregates.

We found that CPY*-mRFP aggregates alone activate both the UPR and the ERSU pathway (Figure 2A,C), although activation was significantly less robust than when induced by Tm (even at 0.5 μg/ml) and was more equivalent to a low/moderate level of ER stress (Figure 2E,F). We found that a majority of class I cells (83%) retained CPY*-mRFP aggregates in the mother and only 17% of daughter cells had inherited the aggregates. At face value, these numbers appear to indicate that CPY*-mRFP aggregates are retained in the mother cells. However, daughter cells lacking the inherited cER should not contain CPY*-mRFP aggregates (Figure 2G). In fact, ER stress induced by CPY*-mRFP aggregate expression blocked cER inheritance in a large population of the class I daughter cells. Thus, when the analysis was restricted to daughter cells that contain the cER, we found that 63% of these cER-positive daughter cells also contained CPY*-mRFP aggregates (Figure 2G). Even for cells with larger buds (class II or III), we observed that CPY*-mRFP localization was dictated by the presence of the cER in the daughter cell. Thus, we conclude that the cellular distribution of CPY*-mRFP aggregates is determined by the location of the cER. Our conclusions are further supported by the observations with GFP-CFTR; we found that GFP-CFTR aggregates did not activate the ERSU pathway, in agreement with the lack of UPR activation (Zhang et al., 2001). If a separate mechanism exists to retain ER protein aggregates in the mother cell irrespective or independently of the ER inheritance block, we would expect that GFP-CFTR aggregates should be retained in the mother cells. However, we found that GFP-CFTR aggregates entered the daughter cells effectively (Figure 2H), and there was no evidence for preferential retention of aggregates in the mother cell. Finally, further support for the hypothesis that the distribution of ER protein aggregates is dictated by the ER inheritance status came from our study of ERSU-deficient *slt2Δ* cells. These cells do not undergo a block in ER inheritance in response to ER stress and accordingly, *slt2Δ* daughter cells contained higher levels of CPY*-mRFP aggregates than do WT CPY*-mRFP-expressing daughter cells (Figure 3).

One notable difference in the behavior of CPY*-mRFP and GFP-CFTR aggregates was that only CPY*-mRFP aggregates induced the ERSU pathway (Figure 2C vs Figure 2D). The aggregates had similar biochemical behavior, as indicated by their detergent insolubility (Figure 1E). Interestingly, GFP-CFTR aggregates were present in almost all of the cER-positive small-budded cells (class I: <2-μm diameter, gray vs yellow bars in Figure 2H), whereas CPY*-mRFP aggregates were present in only about half of cER-positive small-budded cells (gray vs yellow bars in Figure 2G). A similar trend was found after treatment with Tm (Figure 2—figure supplement 1D vs Figure 2G). These data suggest that GFP-CFTR aggregates behave like soluble proteins in the ER lumen, perhaps because of the lack of proteotoxicity. In this regard, analysis of GFP-CFTR aggregates could provide a unique opportunity to monitor the behavior of soluble non-toxic ER luminal proteins in live cells.

We found that the behavior of CPY*-mRFP and GFP-CFTR aggregates in *bud1Δ* cells and WT cells was indistinguishable, indicating that the mother–daughter lateral diffusion barrier established by Bud1 does not play a significant role in either the cER inheritance block or the distribution of ER protein aggregates in response to ER stress. Our data differ from those in a previous study (Clay et al., 2014), which showed an increase in CPY*-GFP foci in *bud1Δ* daughter cells and thus suggested an important role for Bud1 in the retention of CPY*-GFP foci in the mother cell. Paradoxically, that study observed only one or two CPY*-GFP foci in WT daughter cells and up to six CPY*-GFP foci in *bud1Δ* daughter cells. Interestingly, Barral and colleagues argued that the greater abundance of CPY*-GFP foci in the *bud1Δ* daughter cells was due to increased transfer of the soluble form of CPY*-GFP from the mother and subsequent formation of CPY*-GFP aggregates in the daughter cell (Clay et al., 2014). However, there was no direct demonstration that soluble CPY*-GFP levels were in fact increased. During our analysis (n > 100 cells per experiment with at least three independent repeats), we never observed significant differences in the average number of CPY*-mRFP aggregates in WT and *bud1Δ* daughter cells, or even in WT and *bud1Δ* mother cells (data not shown). The reason for the different findings in the two studies is currently not clear. The ERSU pathway functions in at least two different yeast strain backgrounds (W303 and BY4741). Interestingly, *bud1Δ* mother cells appear to have a shorter replicative lifespan than WT mother cells under optimal stress-free growth conditions. If CPY* aggregates are retained in the WT mother cell in order to preserve ER proteostasis in the daughter cell and thus ensure its longevity, one may anticipate that *bud1Δ* mother cells, which contain fewer CPY* aggregates, would have better ER proteostasis and thus live longer than WT mother cells (Clay et al., 2014). Together with other data presented here, our finding that CPY*-mRFP aggregate distribution correlates with the lack of the ER inheritance block in *bud1Δ* cells strengthens the hypothesis that the ERSU pathway regulates the distribution of ER protein aggregates in yeast daughter cells.

Our results suggest a potentially significant difference in the manner in which cells cope with cytoplasmic vs ER protein aggregates. To date, several distinct cytoplasmic protein aggregates have been reported: ubiquitinated cytoplasmic proteins associate with JUNQ, whereas insoluble proteins associate with IPOD upon proteasome inactivation (Kaganovich et al., 2008) or in the presence of amyloid proteins such as Huntingtin with extended polyQ. In cells with functional proteasomes, misfolded proteins dynamically form inclusion bodies called Q-body protein aggregates (Spokoini et al., 2012; Roth and Balch, 2013; Zhou et al., 2014). An interesting feature of these cytoplasmic protein aggregates is their differential subcellular localization; JUNQ associates with the ER/nucleus, IPOD is found next to the vacuole (Kaganovich et al., 2008; Ogrodnik et al., 2014; Polling et al., 2014), and Q-bodies are scattered throughout the cytoplasm (Spokoini et al., 2012; Escusa-Toret et al., 2013; Zhou et al., 2014). These protein aggregates are selectively retained in the mother cell, and a recent study has revealed an intriguing mechanism for retention of Q-body protein aggregates through association with the ER and eventually with the mitochondria (Zhou et al., 2014). We found that fluorescently tagged forms of both CPY* and CFTR aggregates appeared to be scattered throughout the ER. Ultimately, the ERSU pathway blocks ER inheritance and prevents transmission of ER protein aggregates into the daughter cell. The asymmetric distribution of cytosolic protein aggregates and large inclusions such as JUNQ and IPOD is protective and allows the daughter cells to be rejuvenated during each cell division. ER aggregates may similarly be prevented from entering the daughter cell. But during ER stress, ER aggregates are retained by the ERSU mechanism that prevents transfer of the stressed ER. Cytosolic protein aggregates have been reported to associate with the ER, raising the intriguing possibility that the ER functions as a central controller for the distribution of protein aggregates in the cell. Alternatively, the association of cytoplasmic protein aggregates with the ER may somehow induce ER stress and consequently, the ERSU pathway. In this scenario, the ERSU pathway may function as a master regulator for both the ER and cytoplasmic protein aggregates.

Many of the cell cycle checkpoints that ensure accurate DNA replication and genome transmission are restricted to specific stages of the cell cycle (Rhind and Russell, 2012; Yasutis and Kozminski, 2013). By observing asynchronous populations of yeast cells, we found that both class II and class III cells exhibit the block in ER inheritance during ER stress, but it is less pronounced than in class I cells, as we described in our initial report. This finding suggests that ER stress must be recognized early in the cell cycle in order to induce the ERSU pathway. Significantly, ER transfer to the daughter cell had already taken place in many class II and III cells prior to encountering ER stress, and presumably, these populations contribute to the lower number of cells exhibiting the cER inheritance block. Using synchronized cells, we found that cells at later stages of the cell cycle, when the cER is already in the daughter cell at the time of exposure to ER stress, undergo cytokinesis for the first round of the cell cycle but exhibit a block in cER inheritance and cytokinesis during the second round. Thus, the ERSU pathway is effective only when ER stress is sensed at an early stage of the cell cycle and can be ignored until the second cell cycle if the cER was already present in the daughter cell.

Such a mode of ERSU pathway regulation of ER inheritance is rather unusual when compared to cell cycle checkpoints that regulate DNA replication and transmission (Vleugel et al., 2012; Hayashi and Karlseder, 2013). Failure to align chromosomes properly, for example, activates the spindle assembly checkpoint, leading to inhibition of the anaphase-promoting complex and induction of cell death (Chang and Barford, 2014). To our knowledge, the observation that class II and III cells with cER-containing daughter cells proceed normally through the first round of cytokinesis after ER stress, *and that this ‘error’ is not corrected* until the second round, is unprecedented. Results of the FRAP experiments indicate that ER stress was manifested within 30 min of stress induction in both class II or III daughter cells. Thus, these cells must somehow bypass the ERSU pathway-induced cytokinesis block in the first round of division but halt the cell cycle during the next round of cytokinesis. In the case of the replication checkpoints, it would not be possible to lose a chromosome at the first division and then recover during the second cell cycle. In contrast, a functionally stressed ER might be tolerated if the problem is resolved promptly in the next cell cycle. Currently, the molecular mechanisms dictating the decisions by class I, II, and III cells to proceed—or not—through cytokinesis are unknown; the answers to these and other questions raised here await further studies.

## Materials and methods

### FRAP assays

Cells expressing the Kar2/BiP-sfGFP reporter were grown to mid-log phase in filter-sterilized 0.5X YPD (0.5% yeast extract, 1% peptone, and 2% dextrose) and treated with Dimethyl Sulfoxide (DMSO) or tunicamycin (Tm 1 μg/ml) for 3 hr at 30°C. Cells were transferred to 1.6% agarose pads made with 0.5× YPD ± 1 μg/ml Tm and the pads were maintained at 30°C for the duration of the experiment. Photobleaching was achieved with one 0.2-s pulse from a 488-nm argon laser set to 50% power using an Applied Precision optical sectioning microscope (100× 1.65 Apo objective, immersion oil *n* = 1.78 [Cargille Laboratories]) with softWoRx version 3.3.6 (Applied Precision, Issaquah, WA). To compare multiple FRAP events on a single graph, we calculated the fluorescence recovery by determining the relative intensity of the bleached region compared with the unbleached region and defining the bleaching event as 0 and complete recovery as 1 for each photobleached cell. The average fluorescence recovery curves were obtained by averaging the fluorescence recovery values at the same time points for each strain. Images were acquired immediately before and at 6-s intervals after the photobleaching event.

### ER inheritance assays

Cells (WT-MNY1037, *slt2Δ*-MNY1043, *bud1Δ*-MNY2112, these and all other yeast strains used in this study are described in Table 1) expressing Hmg1-GFP were treated with DMSO or Tm (1 μg/ml) unless otherwise indicated, for 3 hr during mid-log phase, imaged with fluorescence microscopy, and scored for the presence or absence of cER in class I, class II, and class III buds. An Axiovert 200M Carl Zeiss Micro-Imaging microscope with a 100× 1.3 NA objective was used as described previously (Babour et al., 2010).

**Table 1.** Yeast strains used in this study **DOI:** http://dx.doi.org/10.7554/eLife.06970.018

Strain name	Genotype	Reference
MNY1037	*MATa, leu2-3,112, trp1-1, can1-100, ura3-1::HMG1-GFP:URA3, ade2-1, his3-11,15::UPRE-lacZ:HIS3*	(Babour et al., 2010)
MNY2215	*MATa, leu2-3,112, trp1-1, can1-100, ura3-1, ade2-1, his3-11,15::HIS3, bar1Δ::LEU2, DsRed-HDEL::ADE2*	(Babour et al., 2010)
MNY1000	*MATa*, *leu2-3,112, trp1-1, can1-100, ura3-1, ade2-1, his3-11,15*	(Cox et al., 1993)
MNY1002	*MATa*, *leu2-3,112, trp1-1, can1-100, ura3-1::HMG1-GFP::URA3, ade2-1, his3-11, bar1Δ::LEU2*	(Bicknell et al., 2007)
MNY2119	*MATa, leu2-3,112, trp1-1, can1-100, ura3-1, ade2-1, his3-11,15::UPRE-lacZ:HIS3 KAR2sfGFP::KanMX*	This study
MNY2702	*MATa*, *leu2-3,112, trp1-1, can1-100, ura3-1::4xUPRE-GFP::URA3, ade2-1, his3-11,15*	This study
MNY1043	*MATa, leu2-3,112, trp1-1, can1-100, ura3-1::HMG1-GFP:URA3, ade2-1, his3-11,15::UPRE-lacZ:HIS3 slt2Δ::KanMX*	(Babour et al., 2010)
MNY2112	*MATa, leu2-3,112, trp1-1, can1-100, ura3-1::HMG1-GFP:URA3, ade2-1, his3-11,15::UPRE-lacZ:HIS3 bud1Δ::KanMX*	This study
MNY2825	*MATa, leu2-3,112, trp1-1, can1-100, ura3-1, ade2-1, his3-11,15::HIS3, bar1Δ::LEU2, DsRed-HDEL::ADE2 slt2Δ::KanMX*	This study

### CPY*-mRFP and GFP-CFTR induction

*S. cerevisiae* strains (WT-MNY1037, *slt2Δ*-MNY1043, *bud1Δ*-MNY2112) were transformed with pFJP10 (pRS425-GAL1-CPY*-mRFP, this and all other plasmids used in this study are described in Table 2). Cells were grown overnight on SCD-Leu with 4% raffinose. Cells were then diluted to OD 0.06, grown to OD 0.25, and then either 2% dextrose (±1 μg/ml Tm) or 2% galactose (±1 μg/ml Tm) was added. Cultures were further incubated at 30°C for 2 hr before cER inheritance, and CPY*-mRFP foci formation were analyzed by fluorescence microscopy. The yeast strains carrying DsRed-HDEL (WT-MNY2215, *slt2Δ*-MNY2825) were transformed with pCU426CUP1/EGFP-CFTR (a gift from Dr Elizabeth Sztul [Fu and Sztul, 2003]). Cells were diluted to OD 0.06, grown to OD 0.25, and then 100 µM copper sulfate ±1 μg/ml Tm was added. Cultures were further incubated at 30°C for 2 hr before cER inheritance, and GFP-CFTR foci formation were analyzed by fluorescence microscopy.

**Table 2.** Plasmids used in this study **DOI:** http://dx.doi.org/10.7554/eLife.06970.019

Plasmid name	Construct	Reference
pFJP1	pFA6a-sfGFP-HDEL::KanMX6	This study
pRH1209	4XUPRE-GFP::URA3 (pJCI86-GFP)	(Cox et al., 1993)
pCU426CUP1/EGFP-CFTR	pRS426-CUP1-EGFP-CFTR	(Fu and Sztul, 2003)
pFJP10	pRS425-GAL1-CPY*-mRFP	This study

### UPRE-GFP strain construction

pRH1209 (*4xUPRE-GFP::URA3*) plasmid (a gift from Dr Randy Hampton) (Hampton et al., 1996) was digested with restriction enzyme StuI and transformed into yeast strain MNY1000 for genomic integration at the URA3 locus to generate strain MNY2702. MNY2702 was then transformed with pFJP10 to induce CPY*-mRFP expression as described above.

### Synchronization

Yeast strain MNY1002 cells were treated with 50 ng/ml α−factor for 2.5 hr, washed twice with an equal volume of fresh YPD containing 1 M sorbitol, diluted to OD 0.25, and then allowed to recover for either 20 min (phase I) or 50 min (phase II) before the addition of 1 μg/ml Tm. Cells were imaged at the indicated time points after Tm addition. For staining of synchronized cells, 200 μg/ml Texas Red-conjugated concanavalin A (TR-ConA, Sigma, St. Louis, MO) was added to the culture for the final 30 min of α−factor treatment, and the culture was incubated in the dark at 30°C with shaking. The cells were washed to remove TR and α−factor and then treated as described above, except that the TR-labeled cell cultures were maintained in the dark. Cells were collected and imaged by fluorescence microscopy at the indicated time points.

### Northern blotting

Cells were synchronized as described above. At the appropriate time points, 20-ml aliquots of cells were collected and flash frozen in liquid nitrogen. Total RNA was extracted as described before (Chawla et al., 2011). Samples of 20 μg of total RNA were separated on a 4.5% agarose gel with 6.7% formaldehyde and transferred to a zeta-probe membrane (Bio-Rad, Hercules, United States) in 10× SCC overnight. After UV-crosslinking, membranes were probed with a radiolabeled HAC1 DNA probe as described in (Chawla et al., 2011).

### Kar2sfGFP strain construction

pFA6a-GFP::KanMX6 (a gift from Jurg Bahler and John Pringle, Addgene plasmid # 39292) (Bahler et al., 1998) was modified by replacing GFP with sfGFP-HDEL. sfGFP-HDEL was PCR amplified from plasmid psfGFP-HDEL (a gift from Dr Erik Snapp) and switched with GFP in pFA6a-GFP::Kan^r^ to generate pFJP1 (pFA6a-sfGFP-HDEL::KanMX6). The plasmid was checked by sequencing. sfGFP-HDEL::KanMX6 was PCR amplified with primers FJP17 (ATAAATTAACAACCTTGAAGCTTCCAGCAGCAAAAATTTTTAACTATTTTATgaattcgagctcgtttaaac) and FJP37(CAGTCTCTATACTCTTCAATG) to tag KAR2 at the genomic locus of strain MNY1004 to generate strain MNY2119 using the Longtine method (Longtine et al., 1998).

### Aggregation assay

Aliquots of 20 ml of cells were collected after 2 hr of induction, washed once with water, and flash frozen in liquid nitrogen. Protein extracts were prepared and the following detergent solubility test was performed (as described in [Alberti et al., 2010]) to characterize the CPY*-mRFP and GFP-CFTR foci observed under the microscope. The cell pellet was resuspended in 600 μl of lysis buffer (50 mM Tris, pH 7.5, 150 mM NaCl, 2 mM ethylenediaminetetraacetic acid (EDTA), 5% glycerol) with protease inhibitors (5 mM phenylmethylsulphonyl fluoride [PMSF], aprotinin, leupeptin, pepstatin A), 50 mM N-ethylmaleimide (NEM), and 25 μM MG132, and then lysed with glass beads at 4°C. A sample of 300 μl of the lysate was mixed with 300 μl of cold detergent-lysis buffer (50 mM Tris, pH 7.0, 150 mM NaCl, 1% Triton X-100, 0.5% deoxycholate, 0.1% sodium dodecyl sulfate, (SDS) and vortexed for 10 s. The remaining 300 μl of sample was diluted with an additional 300 μl of lysis buffer. The crude lysates were centrifuged for 2 min at 800 rcf (4°C) to pellet the cell debris. Supernatant samples (250 μl each) were centrifuged in a TLA 100 rotor for 30 min at 80,000 rpm and 4°C using a TL Beckman ultracentrifuge. The pellet from the RIPA buffer lysate was resuspended in 250 μl of RIPA buffer. The pellet from the lysis buffer only was resuspended in 250 µl of lysis buffer (no detergent). Equal volumes of unfractionated (total), supernatant (S), and pellet (P) samples were incubated in sample buffer containing 2% SDS and 2% β-mercaptoethanol. CPY*-mRFP extracts were heated for 5 min at 95°C, and GFP-CFTR extracts were heated for 20 min at 37°C. Samples were analyzed by SDS-PAGE and Western blotting. CPY*-mRFP was detected with anti-mRFP rat monoclonal antibody (1:1000, cat # ABIN334653, Life Technologies, Carlsbad, CA, United States) and secondary HRP-conjugated anti-rat antibody (Bio-Rad). GFP-CFTR was detected with anti-GFP mouse monoclonal (1:1000, cat # 11814460001, Roche, Basel, Switzerland) and secondary HRP-conjugated anti-mouse antibody (Bio-Rad).
